# Revisiting Pearl’s influenza studies by bootstrapping for forward variable selection with a null factor

**DOI:** 10.1371/journal.pone.0318685

**Published:** 2025-02-25

**Authors:** Roselinde Kessels, Chris Gotwalt, Guido Erreygers

**Affiliations:** 1 School of Business and Economics, Maastricht University, Maastricht, The Netherlands; 2 Department of Economics, University of Antwerp, Antwerp, Belgium; 3 JMP Division, SAS Institute, Cary, North Carolina, United States of America; Italian National Research Council (CNR), ITALY

## Abstract

In 1919 and 1921 Raymond Pearl published four empirical studies on the Spanish Flu epidemic in which he explored the factors that might explain the explosiveness and destructiveness of the epidemic in America’s largest cities. Using partial correlation coefficients he tried to isolate the net effects of the possible explanatory factors, such as general demographic characteristics of the cities and death rates for various diseases, on the variables measuring the severity of the epidemic. Instead of Pearl’s correlation analysis, we apply a bootstrap simulation to forward variable selection with a null factor for generalized linear regression with AICc validation. The null factor or pseudo-variable is a random variable that is independent of the response. The number of times it is included in the model selection simulation provides an important metric for deciding which terms should remain in the model. Our results are largely consistent with Pearl’s conclusions in that the pre-pandemic death rates from organic heart disease and from all causes are most predictive of pandemic explosiveness or severity. However, our results also contain substantive nuances. Our paper contributes to the literature showing that state-of-the-art methodology for variable selection proves useful for historical epidemiology.

## Introduction

Although influenza pandemics have occurred at least since the 16^th^ century, it is mainly in the 19^th^ century that they became widely noticed [1, p. 22–23]. In 1889–1890, for instance, the “Russian Flu” pandemic swept the globe in about four months’ time [2]. The influenza pandemic of 1918–1921, commonly known as the “Spanish Flu” pandemic, was almost instantly perceived as more severe than previous epidemics. The outbreak led to alarming reports and assessments in both medical and general science journals, and provoked discussions about the deadliness of the epidemic [3] and about the cause of the disease [4]. Economists paid surprisingly little attention to the pandemic [5].

By the beginning of 1919, the biostatistician Raymond Pearl (1879–1940), professor of biometry and vital statistics at the School of Hygiene and Public Health of Johns Hopkins University [6,7], was among those who were alarmed by the scale and the seriousness of the pandemic. He urged epidemiologists “to investigate with all possible thoroughness epidemic influenza, to the end of making a better defense next time” [8, p. 1744]. His own contribution to this investigation came in the form of a series of “Influenza Studies”, the first of which he published in August 1919 and the others in 1921. These were statistical studies in which he explored how the severity of the influenza epidemic in the major cities of the US might be related to demographic, geographic and other circumstances. His approach relied heavily on the calculation and interpretation of partial correlation coefficients, a rather cumbersome way of doing multiple correlation analysis. In the second, third, and fourth “Influenza Studies” (published jointly as Pearl [9]) he refined the analysis of his first study, by modifying the variable he had used to measure the “explosiveness” of the epidemic and by introducing a new variable measuring its “destructiveness”.

Pearl’s first study did not go unnoticed. It figured prominently in Winslow and Roger’s [10] work on the epidemic in Connecticut and in Vaughan’s [11] survey of epidemiological research on influenza. Perhaps the main difficulty faced by those who wanted to study the Spanish Flu pandemic while it occurred, was the poor quality of the available data, as pointed out by Edgar Sydenstricker [12]. Together with Wade Frost, his colleague at the United States Public Health Service, Sydenstricker set up surveys in Maryland [13] and reviewed the evidence collected in other countries [14,15]. Frost used these data to compare the Spanish Flu pandemic to the Russian Flu pandemic:

In general, this epidemic has been quite similar to that of 1889–1890 in its early development, first in mild, scattered outbreaks, later in a severe world-wide epidemic; in the rapidity of its spread, and in its high case incidence. It has been notably different in a much higher frequency of pneumonia and consequently much higher mortality, especially among young adults. [16, p. 318]

We now know that the Spanish Flu pandemic was exceptionally severe: the worldwide death toll may have been as high as 50 million people [17]. Probably most casualties occurred in India and China, but Europe and the US were also heavily affected. In the US, as in many other countries, the epidemic came in three different waves, the first two of which occurred in 1918 and the last one in early 1919. The second wave, which reached its peak in the fall of 1918, was the deadliest.

Frost’s observation about the unusual pattern of mortality was also spot on. In the years before the pandemic, the influenza mortality curve had a typical U-shaped pattern: the risk of dying was especially high among the very young and the old. This time, however, the mortality curve showed a W-shaped pattern, with a high mortality rate among young adults. As more and more reliable data became available it was acknowledged that “the relatively high mortality in early adult life was a distinctive and striking feature of the influenza pandemic.” [18, p. 237]

One of the reasons why Pearl wrote three follow-up studies in 1921 is that he had access to more data than in 1919. As a result, he changed the way he measured certain variables. All in all, however, his data set was small and of a highly aggregate nature. By contrast, recent statistical analyses of the 1918–1921 pandemic have been able to exploit richer data sets which are often much more detailed. Examples are the studies by Mamelund [19], on mortality among ethnic minorities in Norway, by Markel et al. [20], on the effect of non-pharmaceutical interventions in US cities, by Mamelund [21], on the influence of geographical isolation, by Grantz et al. [22], on disparities due to sociodemographic factors within Chicago, by Clay, Lewis and Severnini [23], on cross-city variation in 438 US cities, and by Basco, Domènech and Rosés [24], on mortality in Spanish provinces. The review of the literature on health disparities in past influenza pandemics by D’Adamo et al. [25] is quite useful.

In this paper we revisit Pearl’s influenza studies. We consider the same data he had at his disposal, but we go beyond the rather basic statistical analysis he used to find meaningful relationships between the variables. Our main aim is to explore to what extent his results still hold if we apply more advanced statistical techniques. We compare Pearl’s findings from his correlation analyses with the results obtained from modern machine learning methodology involving bootstrapping for forward variable selection with a null factor. To strengthen the selected models, we add a few variables to the study which were not considered by Pearl. It should be noted, however, that our focus is on verifying Pearl’s analyses using modern statistical methodology rather than on identifying all possible variables that might have contributed to the explosiveness and destructiveness of the epidemic in the cities in our samples. We present and discuss our main results, and indicate whether they are in line with Pearl’s findings.

By examining the robustness of Pearl’s influenza studies, our paper contributes to the historical epidemiology literature. It shows the usefulness of applying state-of-the-art methodology for variable selection. Increasingly, modern statistical techniques are used for the analysis of older medical datasets. For instance, O’neil and Sattenspiel [26] developed new agent-based models to study data on the 1918–1919 influenza epidemic in central Manitoba, Qiu et al. [27] applied complex machine-learning models to National Health and Nutrition Examination Survey (NHANES) data, and Ding et al. [28] used a multimodal machine learning framework to predict dementia risk based on data from the Framingham Heart Study. We propose a sound, user-friendly methodology that can be employed by researchers from many disciplines to explore the factors that predict a response in observational data.

## Methods

### Variable selection problem

Establishing significant associations between predictor and response variables from observational studies is a challenging task. In regression analysis on observational data, the predictor variables are generally not orthogonal, which can bias the p-values of the regression coefficients heavily towards zero even if there are no truly important variables under consideration. So, the null distribution of the p-values is no longer uniform and the type-I error rate is completely uncontrolled.

Pearl’s datasets contain relatively many predictors (13 predictors for Study I and 12 predictors for Studies II-IV) of which only a subset are presumptively important. A computationally efficient popular method for detecting important predictor variables from such datasets is forward stepwise selection [29]. This selection procedure starts with an intercept-only model and adds variables one at the time by incorporating at each step the variable that best improves the model fit (as measured by the residual sum of squares, or likelihood) and continuing until all the variables are in the model. The resulting sequence of models, consisting of different subsets of the available predictor variables, are then ranked using a model selection criterion. The model whose combination of incorporated variables optimizes the selection criterion is chosen as the final model.

However, like ordinary regression, forward stepwise selection suffers from inferential problems as demonstrated by Steyerberg et al. [30] for small data sets and by Smith [31] for big data sets. After forward selection has been applied, the usual reported standard errors of the estimates are biased downward, making variables appear more significant than they should be. Forward selection causes the sampling distribution of the individual regression coefficients to have point masses at zero with separate, unknown probabilities, and unknown continuous distributions everywhere else. The point masses at zero are induced by the forward selection algorithm, and the associated probabilities are one minus the selection rates of the variables. These distributions are analytically intractable, as they depend on both the unknown causal relationship between the responses and the truly active predictor variables and the relationship amongst all the predictor variables themselves.

Furthermore, applying forward stepwise selection to multi-correlated data inflates the variances of the estimated regression coefficients, making it harder to identify important predictors. An unimportant variable that is correlated with an important one can then enter the model and effectively block the important variable from entering the model. This is a manifestation of instability where small changes to the data lead to large changes in the model. The elastic net procedure has often been proposed to selecting variables in the case of multicollinearity [32]. However, rather than choosing one variable from a set of correlated input variables, elastic net tends to bring the whole group in, making it less suitable for determining important variables than forward selection.

### Bootstrapping for variable selection with a null factor

We propose a simple solution to the variable selection problem based on the variable selection method introduced by Wu, Boos and Stefanski [33]. More specifically, we add to our set of predictors a pseudo- or phony (noise) variable, called a null factor, which is a random variable that is independent of the response, typically an independent standard normal variable. We use this null factor to calibrate a decision rule for deciding whether or not a variable should be in the model. To that end, we perform a bootstrap simulation with nBoot = 2500 replicates of the data where for each bootstrap replicate we generate new values for the null factor, and apply forward stepwise selection in which we add variables (including the null factor) one at the time to the model and choose the model for that bootstrap replicate based on the smallest value for Akaike’s information criterion corrected for small sample sizes (AICc). The AICc is an estimator of prediction error, the so-called Kullback-Leibler divergence from information theory, which is the distance from the fitted model to the truth. It thereby estimates the relative quality of different models for a given data set. Since the data samples collected by Pearl are very small (*N* = 39 cities for Study I and *N* = 34 cities for Studies II-IV), one must use all the available data for both the model fitting and model selection, and there is a risk that the best fitting model will substantially overfit the data. Fortunately, the AICc validation criterion trades off accuracy of model fit with the number of variables included in the model, leading to parsimonious models. More details on the AICc can be found in Akaike [34] and Hurvich and Tsai [35], and its comparison to the closely related Bayesian information criterion (BIC) in Burnham and Anderson [36], among others.

For the 2500 selected models we calculate the proportion of times the null factor enters the model. Variables that enter as often as or less often than the null factor are ignorable. Variables that enter strictly more often than the null factor are predictive of the response. We consider the 99.9% simulated upper confidence limit (UCL) of the null factor inclusion proportion as a cut-off (corresponding to a 0.1% level of significance α). If *p*_*null*_ represents the proportion of nonzeros of the null factor in the bootstrap simulation, then the 99.9% simulated UCL of this proportion is computed as:


$$\begin{array}{l} p_{null}+NormalQuantile\left(1-0.001\right)*\sqrt{\frac{p_{null}*(1-p_{null})}{2500}},or\\ p_{null}+3*\sqrt{\frac{p_{null}*(1-p_{null})}{2500}}. \end{array}$$

For each bootstrap replicate it is important that the null factor is re-initialized with new random values, otherwise the results are conditional on only one instance of the null factor values and the results are less generalizable.

**Fig 1:**
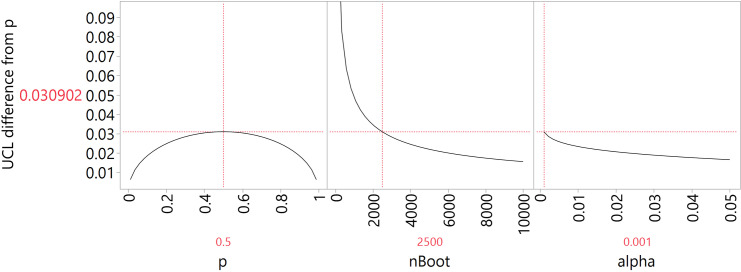
Prediction profiler of the upper margin of an estimated proportion of 0.5, when nBoot is 2500 and level of significance α is 0.001.

What is the motivation for using nBoot = 2500 bootstrap replicates and the 100% * (1 – α) = 99.9% simulated UCL of the null factor inclusion proportion as a cut-off? First, the choice of nBoot = 2500 is a sample size consideration for confidence intervals on a proportion. At that sample size, we know that for any estimated proportion the upper margin does not exceed 0.016 at the 5% significance level (the worst case is when the proportion is 0.5), and ~ 0.01 when the proportion is less than 0.1. The prediction profiler in Fig 1 shows the upper margin or maximum possible interval width (which determines the cut-off for significant differences from the null factor’s entry rate) of an estimated proportion of 0.5, when nBoot is 2500 and level of significance α is 0.001. The profiler illustrates that as long as nBoot is larger than about 1000, the maximum possible interval width does not change much and that the interval widths slowly get narrower after nBoot = 2500. This sample size is kind of a “Goldilocks” sample size. On the one hand, it is not on the boundary of where one has to be concerned about simulation reproducibility; on the other hand, a sample size larger than 2500 does not lead to a huge improvement in precision and repeatability, despite taking longer to run the simulation.

Second, the choice of the significance level α = 0.001 of the simulated UCL of the null factor inclusion proportion is not very critical. At nBoot = 2500 and 100% * (1 – α) = 99.9% the largest possible difference between the upper confidence interval endpoint and the proportion is 0.0309 (see Fig 1) and if we had used 100% * (1 – α) = 95%, it would have been 0.0164. So, it changes by a factor of about 0.5. However, if we were to make this change from α = 0.001 to 0.05, our analysis results would not really change. So in this case, using the level α = 0.001 may not have been necessary. On the other hand, if we declare α = 0.001 in advance, then we are rather sure that the terms that occur more often than the null factor really should be and that it is not a transient fluke of that particular simulation.

Clearly, the general applicability of the nonparametric, resampling-based bootstrap to irregular problems like ours makes it possible to estimate the asymptotic distribution of the forward selection-based regression coefficients via a bootstrap simulation. We can then use the proportion of times a variable enters the model as a surrogate for variable importance. To assess variable importance, we add a null factor to the model to estimate how often it enters the model, just due to chance, and declare that any variable that enters the model as often as or less often than the null factor is definitely not important. We are sidestepping the issues with the standard errors by completely ignoring the (nonzero) magnitudes of the estimates and their reported standard errors in our decision procedure. At the same time, this implies that the variance inflation problem when applying forward selection to multi-correlated data can be circumvented. The resampling-based bootstrapping procedure gives us robust results because we are making changes to the data that preserve the data generating mechanism. This is very similar to bagging [29], except that we propose a decision procedure based on the null factor calibrated proportion rather than simply averaging the predictions.

### Generalized linear regression modelling

Together with the variable selection it is essential to determine a proper distribution for the response variable under study. Both objectives, variable selection and distribution determination, have to be considered simultaneously and can be achieved by minimizing the AICc. The reason why variable selection and distribution determination go hand in hand is that the distribution is the observed consequence of the underlying data “generating” process and therefore we express the mean of the response distribution as a function of our predictor set. The GAMLSS (Generalized Additive Models for Location, Scale and Shape) framework extends this practice to the scale and shape parameters of the distribution as well [37]. For the purpose of this research, we restrict ourselves to two-parameter distributions characterized by location and scale, where the latter is estimated directly with no association to the predictors.

Based on the smallest AICc, we select the most preferred distribution of a generalized linear model for Pearl’s responses given the set of predictors and the null factor. Because the responses are continuous, we choose from the lognormal, Weibull, gamma, Student’s t(5) and normal distributions. Combined with the forward variable selection we refer to our approach as (mean-oriented) generalized regression, to which we apply the bootstrap simulation. Hence, we bootstrap for forward variable selection, for which we can fully rely on the Generalized Regression platform in JMP Pro 17 (SAS Institute, Cary, NC, USA; [38]). To reduce the effect of multicollinearity in the data and to make the estimates comparable, the predictor variables are automatically centered to have a mean of zero and scaled to have a standard deviation of one.

### Data description

The data come from Pearl’s Influenza Studies [8,9] and were provided by the US Bureau of the Census. In Study I Pearl considered a sample of 39 large American cities. Due to a change in variables, the sample size of Studies II-IV was reduced to 34 cities. Pearl initially examined five different epidemicity indices to measure the impact or severity of the influenza epidemic in a city. In Studies I and II he focused on the measure which he believed best reflected “the force of the epidemic explosion in a particular place” [8, p. 1767]. Because of its simplicity, Pearl was most attracted to epidemicity index *I*_5_ in Study I, which he further improved to index *I*_6_ in Study II. Instead of measuring the maximum peak mortality rate for index *I*_5_ during the period when the death rate was higher than the normal death rate, enclosed by the ascending and descending limbs of the mortality curve, the updated index *I*_6_ restricts that period to the ascending limb of the curve, including only the first mortality peak (see the supporting information S1 File for detailed definitions of these indices). In Study III, however, Pearl replaced the “explosiveness” variable *I*_6_ by a “destructiveness” variable representing the excess mortality rate during the epidemic in a city, or “the total number of persons killed by the epidemic” [9: p. 289].

We revisit Pearl’s analyses of these variables using generalized regression with a null factor. In first instance, we consider the same sets of predictor variables Pearl used in his studies. One set consists of demographic variables and another of death rates prior to the pandemic. Table 1 contains an overview of Pearl’s Study I variables with their descriptive statistics. The description of the data from Pearl’s Studies II and III and the full review with our approach can be found in the supporting information S1 File.

**Table 1:** Descriptive statistics of all variables used in Pearl’s Influenza Study I (*N* = 39 cities).

Variable	Mean	Standard deviation	Minimum	Maximum	Description (units)
Epidemicity index *I*_5_	6.78	5.28	0.92	20.51	Peak-time ratio measuring explosiveness of the outbreak in the autumn of 1918 (%)
Population density	15.17	7.65	2.40	30.57	On July 1, 1916 (persons per acre of land area)
Geographical position	721.21	662.51	0.00	2624.00	Straight line distance from Boston (miles)
Age distribution	9.06	2.64	4.76	15.80	Age-constitution index for 1910 (%)
Percentage population growth	40.43	48.94	6.50	245.40	In the decade 1900–1910 (%)
DR (death rate) All causes	15.55	2.24	10.5	19.8	Death rate from all causes for 1916 (number of deaths per 1,000 inhabitants)
DR Pulmonary tuberculosis	147.5	46.33	64.7	262.1	Specific death rates for 1916 (number of deaths per 100,000 inhabitants)
DR Organic heart disease	168.29	39.33	84.7	250.7	
DR Acute nephritis and Bright’s disease	127.36	39.08	70.8	231.1	
DR Influenza	18.80	8.98	4.1	37.4	
DR Pneumonia	158.40	48.62	70.2	331.0	
DR Typhoid fever	12.41	9.76	1.8	43.5	
DR Cancer	97.07	15.19	56.1	133.1	
DR Measles	11.00	10.22	0.0	33.8	

Although we initially looked into the same predictor variables as Pearl did, we obtained different model selection results depending on the variables we included. More specifically, the all-causes death rate can be seen as an aggregate of death rates of all kinds, such as pneumonia, typhoid fever and pulmonary tuberculosis, as represented by the individual death rate variables. The all-causes death rate correlates to a certain degree with most individual death rates (e.g., highest correlation of 0.62 with pulmonary tuberculosis death rate). As such, we applied forward selection with a null factor to Pearl’s predictor variables including and excluding the all-causes death rate variable. This aggregate turned out to be a strong determinant of the epidemicity indices and on top of that, we obtained slightly different model selections when excluding it from the predictor set (see Results). To verify the selected models, and to potentially augment them for relevance, we applied forward selection with a null factor in a second step on the selected variables from Pearl’s set in combination with other potentially relevant 1910 Census variables. We selected these additional variables from the 1910 Census data because Pearl’s set also contained variables from these data. The additional variables are listed in Table 2, along with their descriptive statistics.

**Table 2:** Descriptive statistics of additionally selected 1910 Census variables for Pearl’s Influenza Study I (*N* = 39 cities). Descriptives of these variables for the smaller Pearl’s Influenza Studies II and III are alike (*N* = 34 cities).

Variable	Mean	Standard deviation	Minimum	Maximum	Description (units)
Number of persons to a dwelling	6.40	2.12	4.4	15.6	Housing crowding measure (persons per dwelling)
Percentage of homes owned	30.39	9.06	11.7	47.9	Home ownership rate (%)
School attendance	60.58	4.36	51.0	69.8	Attendance rate in the population 6 to 20 years of age (%)
Illiteracy	4.95	2.58	1.9	13.2	Illiteracy rate in the population 10 years of age and over (%)
Share ages 0–4	9.28	1.14	7.0	11.7	Shares in the population (%)
Share ages 5–14	16.55	1.70	11.9	20.4	
Share ages 15–24	20.27	1.30	18.1	23.4	
Share ages 25–44	34.37	1.99	30.2	40.9	
Share ages 45–64	15.54	1.55	11.7	18.7	
Share ages 65+	3.82	0.73	2.1	5.4	

**Fig 2:**
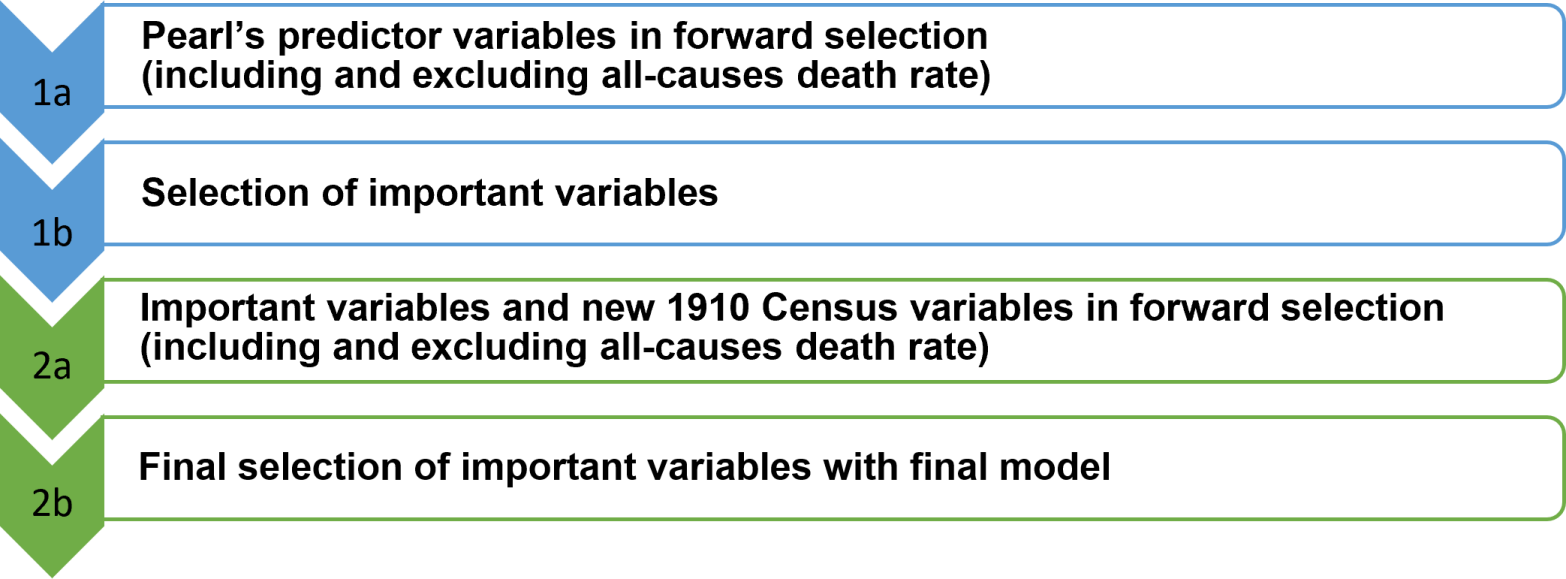
Overview of the two-step variable selection sequence with a null factor.

Among the additional 1910 Census variables, we included six population age shares as a simpler and more direct alternative to Pearl’s age distribution variable, an age-constitution index that we believe is complicated and difficult to interpret. Corresponding to the W-shape pattern of influenza deaths during the epidemic, we only retained the population shares of the ages 0–4, 25–44 and 65+ as proxies for the extreme number of deaths in these age groups. We initially involved all six age shares in our forward selection, but the three age shares mentioned were sufficient to capture all relevant effects. To summarize, Fig 2 illustrates the overall two-step variable selection sequence with a null factor.

## Results

### Specification of the generalized regression

We simultaneously address the choice of the distribution of the response variable, the epidemicity index *I*_5_, and the selection of the predictor variables. We do so by evaluating the fit of commonly used distributions where the mean of the response variable is a function of the predictor variables including the null factor. Using forward variable selection, we retain both the response distribution and model variables that provide the smallest AICc.

**Fig 3:**
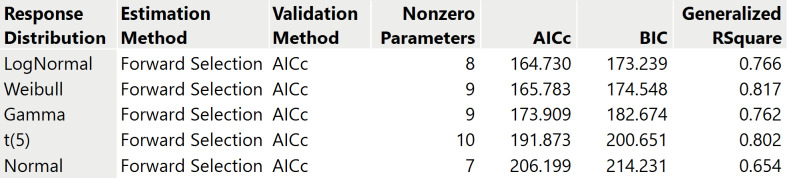
Comparison of model and response distribution of Pearl’s epidemicity index *I*_5_ using forward selection with AICc validation on Pearl’s predictor variables and the null factor for one bootstrap data replicate.

**Fig 4:**
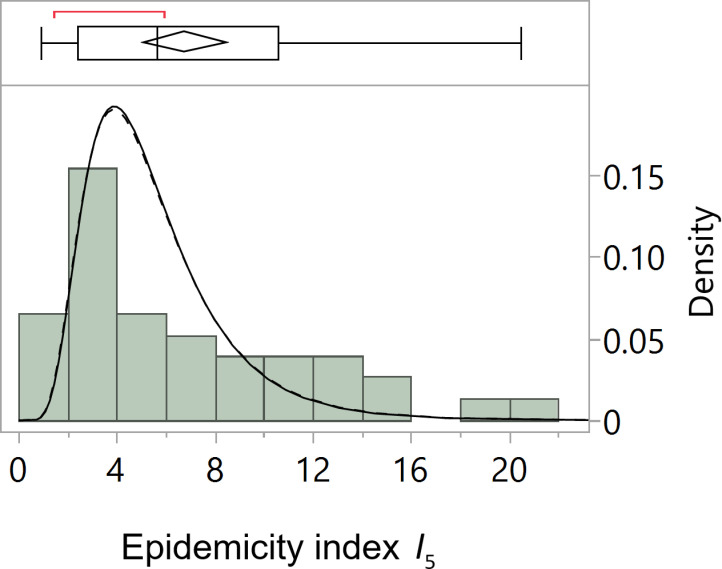
Histogram with outlier box plot of Pearl’s epidemicity index *I*_5_ with fit to the estimated lognormal distributions of Table 7 **using the means of the selected predi***c***tors.** *Note:* The solid line corresponds to the estimated distribution including the all-causes death rate and the dashed line excluding it. The bracket outside the outlier box identifies the shortest half or the smallest interval with 50% of the observations, and the confidence diamond inside contains the mean with its the lower and upper 95% confidence limits.

**Fig 5:**
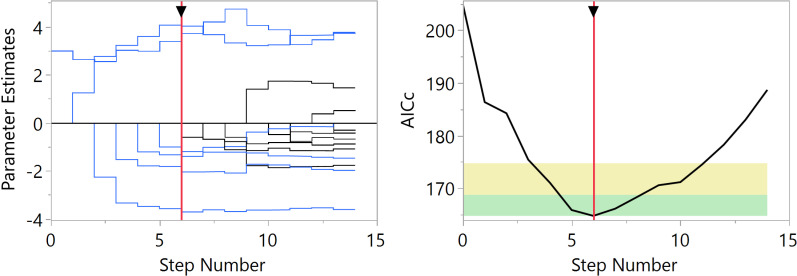
Forward selection solution path showing for each step the magnitude of scaled parameter estimates for the selected model variables from one bootstrap data replicate (left) with corresponding minimum AICc value (right). *Note:* The green zone in the AICc plot (right) is the interval [minAICc, minAICc + 4] and identifies models for which there is strong evidence that they are comparable to the minimum AICc model. The yellow zone is the interval ]minAICc + 4, minAICc + 10] and identifies models for which there is weak evidence that they are comparable to the minimum AICc model.

For *I*_5_ the lognormal distribution turns out to be the better distribution to use throughout our analyses. Starting from the set of Pearl’s predictor variables and the null factor for one bootstrap replicate, Fig 3 shows the ranking of the different distributions associated with the number of nonzero parameters in the selected or “best” models in terms of the AICc. Overall, the lognormal distribution is preferred to model *I*_5_ in function of the predictors (see the supporting information S1 File for a formal description of the lognormal distribution). Accordingly, the distribution of the index, shown in Fig 4, is strictly positive and right-skewed with a median of 5.6 and a mean of 6.8 (see also Table 1). To select the “best” model under the lognormal distribution for *I*_5_, Fig 5 presents the solution path for each step of the forward selection based on one bootstrap replicate showing the magnitude of scaled parameter estimates for the variables and corresponding minimum AICc. The estimates of the original variables appear in Table 3, where a zero estimate indicates that the variable was not included in the model for that bootstrap replicate. Table 4 contains an overview of the estimates for five variables from 16 bootstrap replicates.

**Table 3:** Lognormal regression model for epidemicity index *I*_5_ (Study I) from using forward stepwise selection based on one data set replicate, showing the parameter estimates for the original predictors and the null factor. A value of 0 means the variable is excluded from the model.

Model term	Estimate	Std Error	P-value Chi-square test
Population density	0	0	1
Geographical position	0	0	1
Age distribution	0	0	1
Percentage population growth	0	0	1
DR (death rate) All causes	0.2605	0.0342	<0.0001
DR Pulmonary tuberculosis	0	0	1
DR Organic heart disease	0.0169	0.0024	<.0001
DR Acute nephritis and Bright’s disease	−0.0048	0.0022	0.0330
DR Influenza	0	0	1
DR Pneumonia	0	0	1
DR Typhoid fever	−0.0307	0.008	0.0001
DR Cancer	−0.0396	0.0065	<.0001
DR Measles	0	0	1
NULL FACTOR	−0.2384	0.0689	0.0005
Constant	−0.4815	0.5794	0.406
Scale lognormal distribution	0.3622	0.0410	<.0001

**Table 4:** Snapshot of the bootstrap estimates of four predictors and the null factor for the first 16 data set replicates and epidemicity index *I*_5_ as response (Study I). A value of 0 means the variable is excluded from the model.

Data set replicate	Geographical position	DR All causes	DR Organic heart disease	DR Influenza	NULL FACTOR
1	0	0.2605	0.0169	0	−0.2384
2	0	0.1991	0.0113	−0.0224	0
3	0	0.5079	0.0052	−0.0412	0
4	0	0.2068	0.0113	0	0
5	0	0.4460	0	−0.0213	−0.1152
6	0	0.0397	0.0021	0	−0.2207
7	0	0.1358	0.0100	0	0
8	0	0.2924	0	−0.0325	−0.1044
9	−0.0003	0	0.0207	0	0
10	−0.0009	0.3333	0	−0.0256	0
11	0	0.0566	0.0094	0	−0.0334
12	−0.0005	0.4517	0	−0.0579	0.1571
13	−0.0001	0.0560	0.0055	0	0
14	−0.0004	0.0516	0.0183	0	−0.0960
15	0	0.0898	0.0166	−0.0280	0
16	−0.0004	0.3079	0	−0.0235	−0.1533

### Selected variables for epidemicity index *I*_5_

Pearl’s zero-order correlation coefficients between *I*_5_ and the predictors can be found in the left panel of Table 5, whereas the middle and right panels of this table contain the initial forward selections on Pearl’s predictors including and excluding the all-causes death rate.

**Table 5:** Results for epidemicity index *I*_5_ (Study I) using Pearl’s correlation analysis (left) and bootstrap simulation on the lognormal forward selection model with the null factor and AICc validation (middle and right). Important or selected variables appear in bold on top.

Pearl’s Study I		Forward selection lognormal AICc model simulation			
		DR All causes included (cfr. Pearl)		DR All causes excluded	
	Correlation coefficient		% nonzero in simulation		% nonzero in simulation
**DR All causes**	**0.661**	**DR Organic heart disease**	**0.863**	**DR Organic heart disease**	0.977
**DR Organic heart disease**	**0.567**	**DR All causes**	**0.814**	**DR Pneumonia**	0.940
**DR Pulmonary tuberculosis**	**0.525**	**DR Pneumonia**	**0.554**	**DR Pulmonary tuberculosis**	0.612
**DR Acute nephritis and Bright’s disease**	**0.507**	DR Cancer	0.484	**Geographical position**	0.450
DR Pneumonia	0.388	DR Influenza	0.449	DR Typhoid fever	0.414
Geographical position	−0.348	DR Measles	0.446	DR Cancer	0.391
Percentage population growth	−0.327	NULL FACTOR	0.440	NULL FACTOR	0.382
DR Influenza	0.287	Geographical position	0.386	DR Influenza	0.332
Age distribution	−0.262	DR Pulmonary tuberculosis	0.378	DR Measles	0.280
DR Cancer	0.198	Population density	0.378	Population density	0.220
DR Typhoid fever	0.176	DR Typhoid fever	0.293	Percentage pop growth	0.217
Population density	0.092	DR Acute nephritis and Bright’s disease	0.278	DR Acute nephritis and Bright’s disease	0.209
DR Measles	0.069	Age distribution	0.239	Age distribution	0.198
		Percentage pop growth	0.203		
		**NULL FACTOR 99.9% (Sim) Upper CL**	**0.470**	**NULL FACTOR 99.9% (Sim) Upper CL**	**0.412**

*Note:* Variables in grey enter about as often as the null factor in the selected models from the 2500 bootstrap replicates. Their inclusion proportions are close to the 99.9% simulated upper confidence limit that is considered as a cut-off.

#### Pearl’s correlation analysis.

For epidemicity index *I*_5_ examined in Study I, Pearl observed the highest correlation coefficient with the all-causes death rate, and concluded that “an essential factor in determining the degree of explosiveness of the outbreak of epidemic influenza in a particular city was the normal mortality conditions prevailing in that city.” [8, p. 1781]. Cities with a relatively high normal death rate for 1916 had a relatively severe and explosive mortality during the pandemic, whereas cities with a low normal death rate had a low and more gradual increase in mortality.

Similarly as for the all-causes death rate, Pearl obtained high correlation coefficients between *I*_5_ and the death rates from organic heart disease, pulmonary tuberculosis and acute nephritis and Bright’s disease (kidney disease). Moreover, Pearl investigated whether these high correlations were still observed after correcting for differences in the age distribution of the population as well as differences in the geographical position of the cities, measured by a ‘straight line distance from Boston’. Pearl accounted for geographical position in his calculations because he found a small (negative) correlation with the explosiveness of epidemic mortality, though he did not have sufficient evidence to conclude that the force of the epidemic diminished for cities further away from Boston. As such, Pearl computed the partial or net correlations between the epidemicity index and the death rates from each of the three diseases, for a constant age distribution of the population and constant geographical position. The values he obtained were 0.609, 0.594 and 0.510 for pulmonary tuberculosis, organic heart disease and acute nephritis and Bright’s disease, respectively, and all larger than the uncorrected zero-order correlations. He therefore concluded that the pre-pandemic normal death rates, particularly those from pulmonary tuberculosis, diseases of the heart and of the kidneys were most important in determining the explosiveness of the outbreak of the second wave of the Spanish Flu pandemic.

#### Forward variable selections with a null factor.

Regarding the initial forward selections on Pearl’s predictors, the proportion of times the null factor enters the models from the 2500 bootstrap replicates is quite high and amounts to 44% when including the all-causes death rate and to 38.2% when excluding it. This demonstrates how alarmingly easy it is for unimportant variables to pop up as significant or to be picked out by the variable selection algorithm. The inclusion proportions of the null factor can be considered as false entry or discovery rates and their 99.9% simulated upper confidence limits as cut-off or threshold values to distinguish the important variables from the unimportant ones.

When we consider the all-causes death rate in the variable selection algorithm, this variable enters the model in 81.4% of the bootstrap replicates, which is far beyond the threshold of 47%. Therefore, the all-causes death rate is highly significant. The inclusion proportion of the death rate from organic heart disease is equal to 86.3% and thus slightly larger than that of the all-causes death rate. The death rate from organic heart disease even ranks first in the list of selected predictors and seems to be most predictive of pandemic explosiveness or severity. A third and last significant variable is the death rate from pneumonia, but its inclusion proportion is smaller, amounting to 55.4%. The result that the pneumonia death rate is also important is not supported by Pearl’s findings, although there was some speculation of a possible association between the influenza virus and bacterial pneumonia during the pandemic [8]. For persons who died after having had influenza during the pandemic it was in fact very difficult to correctly diagnose the terminal cause of death as either “pneumonia” or “influenza”. For that reason, the official statistics for these diseases during the pandemic may not be so reliable.

The all-causes death rate turns out to be important in explaining pandemic explosiveness, although it may hamper other death rate variables from entering the models in the bootstrap simulation. Therefore, we excluded the all-causes death rate from the variable selection algorithm. Our results support the inclusion of the pulmonary tuberculosis death rate, which entered the models in 61.2% of the time, and to a smaller extent geographical position, which was selected 45% of the time. The death rate from organic heart disease was still most important, with an inclusion proportion of 97.7%, closely followed by the pneumonia death rate, with a proportion of 94%. Hence, the all-causes death rate was masking the pulmonary tuberculosis death rate and geographical position. Also Pearl emphasized the importance of the pulmonary tuberculosis death rate, but a tentative explanation for this was only later given by Noymer [39,40]. He discussed that before the pandemic, tuberculosis death rates were the highest, and may therefore have been captured by the all-causes death rates, whereas pneumonia and influenza killed far fewer people. With the pandemic, death rates for pneumonia and influenza greatly exceeded those for tuberculosis, which in fact hastened the decline of tuberculosis in the US [40]. Evidence in favour of the importance of geographical position is generally absent, although Pearl found a small negative correlation coefficient with pandemic explosiveness.

In the second step of our variable selection sequence, we considered the important variables from Pearl’s predictor set and the new 1910 Census variables. The resulting forward selections appear in Table 6. When we involved the all-causes death rate in the models, the share of infant ages 0–4 becomes important, while the pneumonia death rate loses its significance. This result can be explained by the very high infant mortality rate due to a combination of influenza and pneumonia in 1918, as shown by the age-specific W-shaped mortality curve in the US at that time. For the analysis without the all-causes death rate, the share of infant ages 0–4 also becomes important, but at the expense of geographical position. So it seems we can safely ignore geographical position, which is consistent with Pearl’s poor statistical evidence for this variable, but also with his doubts about whether the ‘straight line distance from Boston’ is an appropriate indicator of geographical position [8,9].

**Table 6:** Extension of the results for epidemicity index *I*_5_ (Study I) using the important variables from Table 5 and additional 1910 Census variables (from Table 2) in bootstrapping the lognormal forward selection model with the null factor and AICc validation. Important variables appear in bold on top.

Forward selection lognormal AICc model simulation with additional 1910 Census variables			
DR All causes included (cfr. Pearl)		DR All causes excluded	
	% nonzero in simulation		% nonzero in simulation
**DR Organic heart disease**	**0.980**	**DR Organic heart disease**	0.994
**DR All causes**	**0.858**	**DR Pneumonia**	0.816
**Share ages 0–4**	**0.734**	**DR Pulmonary tuberculosis**	0.598
NULL FACTOR	0.354	**Share ages 0–4**	0.464
School attendance	0.304	Illiteracy	0.381
DR Pneumonia	0.263	NULL FACTOR	0.368
Share ages 25–44	0.251	Share ages 25–44	0.361
Persons to a dwelling	0.235	Percentage of homes owned	0.342
Percentage of homes owned	0.220	School attendance	0.330
Illiteracy	0.156	Geographical position	0.318
Share ages 65+	0.092	Persons to a dwelling	0.286
		Share ages 65+	0.096
**NULL FACTOR 99.9% (Sim) Upper CL**	**0.383**	**NULL FACTOR 99.9% (Sim) Upper CL**	**0.397**

**Table 7:** **Selected lognormal regression models for epidemicity index** *I*_5_ **(Study I) based on the original predictors.**

Model term	Estimate	Std Error	*P*-value Chi-square test
**DR All causes included (cfr. Pearl)**			
DR Organic heart disease	0.0116	0.0024	<0.0001
DR All causes	0.1700	0.0395	<0.0001
Share ages 0–4	0.2423	0.0741	0.0011
Constant	−5.2638	0.9135	<0.0001
Scale lognormal distribution	0.4756	0.0538	<0.0001
AICc	188.369		
BIC	194.869		
Generalized R^2^	0.682		
**DR All causes excluded**			
DR Organic heart disease	0.0125	0.0024	<0.0001
DR Pneumonia	0.0049	0.0019	0.0108
DR Pulmonary tuberculosis	0.0056	0.0019	0.0026
Share ages 0–4	0.2034	0.0876	0.0202
Constant	−4.0034	0.9347	<0.0001
Scale lognormal distribution	0.4821	0.0546	<0.0001
AICc	192.240		
BIC	199.597		
Generalized R^2^	0.673		

The final selection models for the distributional mean parameters are contained in Table 7 and visualized using the means of the predictors as input in Fig 4. The model with the all-causes death rate shows that a unit increase in the death rate from organic heart disease, from all causes and the share of infant ages 0–4 increases *I*_5_ by 1.17% (i.e., exp(0.0116) – 1), 18.53% and 27.42%, respectively. Using the means of the predictors (see Table 1) as input to this model yields an average prediction for *I*_5_ of 5.479, with location of 1.588 and scale of 0.476. The corresponding lognormal distribution is plotted by the solid line in Fig 4. The model without the all-causes death rate shows that a unit increase in the death rate from organic heart disease, from pneumonia, pulmonary tuberculosis, and the share of infant ages 0–4 increases *I*_5_ by 1.26% (i.e., exp(0.0125) – 1), 0.49%, 0.56% and 22.56%, respectively. Using the means of the predictors (see Table 1) as input to this model yields an average prediction of 5.496, with location of 1.588 and scale of 0.482. The corresponding lognormal distribution is plotted by the dashed line in Fig 4. The two model distributions largely overlap, with similar prediction performance statistics pointing slightly in favor of the model with the all-causes death rate.

## Discussion

In the four empirical studies of Pearl [8,9] on the Spanish Flu pandemic in America’s largest cities, the sample sizes of the data sets are quite small compared to the number of potential explanatory variables. To reveal which predictors are important for the responses, it is vital to assure that most of the discoveries are indeed true and replicable. Inspired by the work of Wu, Boos and Stefanski [33], we revisited Pearl’s correlation analyses by adding a null factor or noise variable to the list of predictors and by bootstrapping for forward variable selection in generalized linear regression. All variables that entered the model more often than the null factor cut-off were considered significant, whereas the others were not. The ignorable variables were either truly not predictive, or there was not enough information to declare them predictive.

Using this null factor approach to determine the false discovery rate in Pearl’s data sets led to more credible conclusions than Pearl’s. We find that our results largely correspond with Pearl’s conclusions in the sense that the pre-pandemic death rates from organic heart disease and from all causes are most predictive of the explosiveness or severity of the pandemic. However, our results also contain substantive nuances. Unlike the conclusions of Pearl’s first study, we were able to identify the pre-pandemic pneumonia death rate as significant, and the pre-pandemic death rate from acute nephritis and Bright’s disease as insignificant. In his second study, Pearl underestimated the importance of population density for explaining the pandemic’s explosiveness, and in his third study, he overestimated the importance of the pre-pandemic all-causes death rate as a predictor of the pandemic’s destructiveness.

Winslow and Rogers [10] obtained a similar strong correlation between the pre-pandemic death rates and the 1918 mortality rates in their study on the Spanish Flu pandemic in Connecticut. Later studies for the US by Grantz et al. [22] and Clay, Lewis and Severnini [23] provided similar evidence. Low levels of health greatly impacted pandemic severity. Whereas Grantz et al. [22] emphasised illiteracy as the closest proxy for population health, Clay, Lewis and Severnini [23] used the pre-pandemic infant mortality rate (in the years 1915 and 1916) as a measure of population health. In this respect, infant deaths directly reflect existing health conditions (diseases, pollution and nutrition), unlike adult mortality, which is linked to an accumulation of lifetime exposure. As far as the effect of the pre-pandemic pneumonia death rate is concerned, we can align our last result with the research of Acuna-Soto, Viboud, and Chowell [41], and references therein, who showed that for US cities pre-pandemic pneumonia death rates partly explained influenza mortality rates during the pandemic.

To strengthen our results, we looked into the effect of some additional 1910 Census variables on the response variables. For *I*_5_ we found the share of infant ages 0–4 to be important, which is consistent with the very high infant mortality rate due to a combination of influenza and pneumonia in 1918. For *I*_6_ and especially the destructiveness variable (see the supporting information S1 File for detailed results), the illiteracy rate was influential. Cities with more illiterate residents led to slightly higher values for *I*_6_ and destructiveness. Lastly, the share of young adults in the 25–44 age group mattered to a small extent to explain destructiveness. However, we did not find enough evidence to fully model the age-specific W-shaped mortality curve during the pandemic, which also presents high mortality rates for the elderly.

In the literature on the Spanish Flu pandemic, other important variables emerge that explain cross-city variation in epidemic explosiveness and destructiveness, but which we did not consider in our analysis. For example, early and sustained implementation of non-pharmaceutical interventions during the pandemic, including school closure, cancellation of public gatherings, and isolation and quarantine, played a crucial role in delaying and reducing peak mortality in cities [20,42]. Also, cities with high levels of air pollution, as measured by coal-fired capacity, experienced significant higher excess mortality rates [23]. As such, the aim of the paper was not to capture all determinants of the epidemicity indices and excess mortality rates, but to verify Pearl’s correlation analyses with bootstrapping for forward variable selection with a null factor.

When it comes to recommendations for future pandemics, we have to keep in mind that pandemics differ from each other. The Spanish flu pandemic stands out because of its W-shaped mortality curve, showing a high mortality rate among young adults. But what is important during every pandemic is clear and timely communication in lay language with the public. Since “illiteracy” turns out to be an important factor in explaining epidemicity index *I*_6_ and destructiveness, it is therefore essential to inform and educate people on how to deal with a pandemic and what to do in case of infection. Moreover, people with underlying medical conditions, such as heart conditions, should be extra careful when making contact with other people.

As far as methodological aspects are concerned, the null factor or pseudo-variable approach in combination with generalized regression is straightforward and easy-to-use, but requires the predictor variables not to be highly correlated. A more general and flexible variable selection approach that accounts for the correlation structure in the predictor variables is called the knockoff filter, or knockoffs, created by Barber and Candès [43]. The method consists of adding terms (called knockoffs) that mimic the correlation structure amongst the predictors. It is more sophisticated in the sense that it has more proven theory behind it to support that it really controls the false discovery rate. When the knockoff method is used instead of the null factor approach, the power to detect important variables is larger, but also decreases substantially as the degree of multicollinearity increases. In that sense, no method can withstand high degrees of multicollinearity. Applying the knockoff method to the analysis of the Pearl data sets would be an interesting topic for future research.

Another avenue worth exploring is to apply Self Validated Ensemble Modeling (SVEM) to Pearl’s data sets. Lemkus et al. [44] developed this technique for the analysis of small sets of experimental data with the aim of predicting future observations. Traditional analysis techniques do not guarantee high prediction performance because of the small sample sizes that prevent partitioning the data into training and validation sets, a strategy that is used in machine learning to improve out-of-sample prediction. SVEM, however, incorporates all data both for training and validation by applying a weighting scheme on the data. This scheme is based on the fractional random weight bootstrap that mimics data partitioning by assigning anti-correlated training and validation weights to each observation [45]. So instead of bootstrapping for forward variable selection as we have done in this work, Pearl’s observational data sets could be analyzed using SVEM with forward selection and other modelling approaches.

## Supporting information

S1 File Appendix to “Revisiting Pearl’s influenza studies by bootstrapping for forward variable selection with a null factor”. (DOCX)
