# Supplementary material for: Revisiting Pearl’s influenza studies by bootstrapping for forward variable selection with a null factor
Source: PLoS One. 2025 Feb 25;20(2):e0318685. doi: 10.1371/journal.pone.0318685 (PMC11856291; doi:10.1371/journal.pone.0318685)
Supplement: S1 File — (DOCX) [file pone.0318685.s001.docx]

Supporting Information

Appendix to “Revisiting Pearl’s Influenza Studies by Bootstrapping for Forward Variable Selection with a Null Factor”

**Methods**

**Data description of Pearl’s Studies I-III**

Pearl’s Studies II-III mostly comprise updated, more refined variables from his first study to more accurately capture the relationships. In this way, he wanted to respond to the criticism he had received about the low quality or construct validity of the data. **Table A1** contains an overview of Pearl’s Studies II-III variables with their descriptive statistics.

In Study I Pearl defined five epidemicity indices to differentiate mortality curves in cities with a single very sharp peak from those that are long, low and flat. Pearl analysed his fifth specification, epidemicity index *I*_5_, which he further developed to index *I*_6_ in Study II. Given their definitions, he called these indices peak-time ratios:

$I_{5}=\frac{P-M'}{T}\text{ }$and $I_{6}=\frac{P-M'}{T'}$,

where *P* is the maximum peak mortality rate observed during the duration *T* (or alternatively $T'$) of the epidemic and $M'$ is the mean death rate in the period from July 6, 1918, to the outbreak of the epidemic. The duration *T*, adopted in *I*_5_, started “when the mortality curve for that city first passed outside the range of fluctuation exhibited by the curve between the week ending July 6, 1918, and the end of the week immediately preceding the epidemic rise of the curve.” The mortality of the outbreak ended “when the curve again passed within the same range of fluctuation.” [1, p. 276] In other words, *T* stands for the number of weeks during which the death rate was higher than the normal death rate for a city in its first continuous outbreak of influenza mortality. This includes, however, both the ascending and descending limbs of the mortality curve, which was criticized for not strictly measuring the explosiveness of the epidemic outbreak. In Study II Pearl addressed the criticism and only considered the ascending limb of the curve. Hence, he adjusted the duration into $T'$ which ends “in the week in which the mortality curve attained its first epidemic peak” [1, p. 276], and referred to the modified epidemicity index as *I*_6_.

**Table A1.** **Descriptive statistics of all variables used in Pearl’s Influenza Studies II and III (*N* = 34 cities).**

| Variable | Mean | Standard deviation | Minimum | Maximum | Description (units) |
| --- | --- | --- | --- | --- | --- |
| Epidemicity index *I*_6_ (Study II) | 18.89 | 11.92 | 2.5 | 47.9 | Modified peak-time ratio measuring outbreak explosiveness in the autumn of 1918 (%) |
| Destructiveness (Study III) | 4.75 | 1.79 | 1.5 | 8.0 | 25-week excess mortality rate from September 8, 1918 to March 1, 1919 compared to 1917 deaths for the corresponding months (number of excess deaths per 1,000 inhabitants) |
| Age distribution | -42.10 | 20.34 | -82.71 | -10.73 | Modified age-constitution index for 1910 (%) |
| Sex ratio | 99.81 | 7.82 | 89.60 | 131.60 | Males per 100 females for 1910 (number) |
| Population density  Latitude  Longitude | 15.93  40.17  84.33 | 7.85  3.19  13.39 | 2.49  29.97  71.06 | 30.57  44.97  122.43 | On July 1, 1916 (persons per acre of land area)  North-south location (degrees from Equator)  East-west location (degrees from Greenwich meridian) |
| Percentage population growth | 35.46 | 37.88 | 6.50 | 211.50 | In the decade 1900-1910 (%) |
| DR All causes | 1524.59 | 216.69 | 1082.8 | 1983 | Death rates averaged over 1915-1917 (number of deaths per 100,000 inhabitants) |
| DR Pulmonary tuberculosis | 148.65 | 42.11 | 76.3 | 270.9 |
| DR Organic heart disease | 171.93 | 36.35 | 100.7 | 237.2 |
| DR Acute nephritis and Bright’s disease | 125.44 | 39.00 | 73.6 | 245.6 |
| DR Typhoid fever | 10.65 | 6.49 | 2.7 | 30 |
| DR Cancer | 98.70 | 15.43 | 66.5 | 138 |

**Results**

**Specification of the generalized regressions**

On the whole, the lognormal distribution is chosen not only to model the epidemicity index *I*_5_ but also the updated index *I*_6_ in function of the predictors. (Since these indices represent peak rates of excess mortality, it is interesting to point out that Urenda et al. [2] argued that the peak immunity levels of people who recovered from COVID-19 also follow a lognormal distribution.) To model the destructiveness variable as a function of the predictor set, the AICc points toward the normal distribution as the better distribution to fit throughout our analyses. This implies that also the residuals follow a normal distribution as shown in the normal quantile plot of **Fig A1**. The normal distribution to explain excess mortality rates during the Spanish Flu pandemic has been used before in a larger study by Clay, Lewis and Severnini [3] who explored 438 US cities.

Similarly to the epidemicity index *I*_5_, index *I*_6_ has a strictly positive, right-skewed distribution with a median of 16.4 that is smaller than the mean of 18.9, as shown in **Table A1** with distribution visualized in **Fig A2a**. Given the predictor set, a lognormal distribution is appropriate. A random variable *Y* is said to have a lognormal distribution with location µ and scale σ if log(*Y*) is normally distributed with mean µ and standard deviation σ. We assume the natural logarithm although other bases can be used as well. The probability density function of *Y* has the form

$$f_{Y}\left(y;\mu,\sigma\right)=\frac{1}{y}\frac{1}{\sigma\sqrt{2\pi}}\exp\left(-\frac{\left(\log\left(y\right)-\mu\right)^{2}}{2\sigma^{2}}\right)\text{, }y>0$$

where the mean of *Y* depends on a set of *p* predictors $X_{1},\ldots,X_{p}$ and is defined as

$E\left(Y\right)=\exp\left(\mu+\frac{\sigma^{2}}{2}\right)=\exp\left(\beta_{0}+\beta_{1}X_{1}+\ldots+\beta_{p}X_{p}+\frac{\sigma^{2}}{2}\right)$.

The regression coefficients $\beta_{0},\ldots,\beta_{p}$ and the squared scale $\sigma^{2}$ are estimated using maximum likelihood. The exponential in the expression ensures that the mean of *Y* is nonnegative. The constant is represented by $\exp\left(\beta_{0}+\sigma^{2}/2\right)$ and the interpretation of the slope coefficients $\beta_{i}\text{ }\left(i=1,\ldots,p\right)$ is that for every unit increase in *X_i_* the predicted value of *Y* increases by $100\left(\exp\left(\beta_{i}\right)-1\right)$percent.

**Fig A1.** **Normal quantile plot of the residuals of Pearl’s destructiveness variable estimated from normal forward selection with AICc validation on Pearl’s predictor variables and the null factor for one bootstrap data replicate.**

**Fig A2.** **Histograms with outlier box plots of (a) Pearl’s epidemicity index *I*_6_ with fit to the estimated lognormal distributions of Table A2c and (b) Pearl’s destructiveness variable with fit to the estimated normal distribution of Table A3c, using the means of the selected predictors.**

(a) Epidemicity index *I*_6_ (b) Destructiveness

*Note:* The solid line in panel (a) corresponds to the estimated distribution including the all-causes death rate and the dashed lines in both panels correspond to the estimated distributions excluding it. The all-causes death rate is irrelevant as a predictor in panel (b). The bracket outside the outlier boxes identifies the shortest half or the smallest interval with 50% of the observations, and the confidence diamond inside contains the mean with its the lower and upper 95% confidence limits.

**Epidemicity index *I*_6_**

*Pearl’s correlation analysis*

In his follow-up Study II, Pearl examined correlations between epidemicity index *I*_6_ and a revised predictor set shown in **Table A1**. The zero-order correlation coefficients appear in the left panel of **Table A2a**. But specifically, Pearl calculated sixth-order partial correlation coefficients between the index and the (revised) normal death rates from the various diseases holding all demographic factors constant. Results confirmed the importance of the death rates from all causes and from organic heart disease because these remained highly correlated with the index under all circumstances. According to Winslow and Grove [4], this result remains perhaps Pearl’s most outstanding discovery. However, the fairly high correlation for the death rates from pulmonary tuberculosis and acute nephritis and Bright’s disease suggested by the zero-order correlation coefficients of 0.578 and 0.447, substantially diminished given that the partial correlation coefficients for constant demographic factors were only 0.389 and 0.307. Hence, the only salient determinant of the epidemic’s explosiveness was the normal death rate from organic heart disease.

*Forward variable selections with a null factor*

Similarly to Pearl’s results, the forward selections on Pearl’s predictors including and excluding the all-causes death rate, presented in the middle and right panels of **Table A2a**, reveal that the death rate from organic heart disease is most important in explaining epidemic explosiveness. This variable enters the model in most bootstrap replicates (in 87.1% and 96.4% of the replicates with and without the all-causes death rate). Also the all-causes death rate turns out to be highly significant when considered in the simulation, evidenced by an inclusion proportion of 83.4%. Different from Pearl’s results is that population density seems to impact *I*_6_. The inclusion proportions of this variable in the simulations with and without the all-causes death rate are 74.8% and 63.5%, and largely exceed the thresholds of 54.1% and 48.3%. Because *I*_6_ purely looks at the upsurge of the epidemic ‘outbreak’, the association with population density is not surprising. This result is consistent with Grantz et al. [5] who found a significant association between influenza virus transmissibility and population density for Chicago in 1918. On the other hand, the impact of population density on influenza mortality rates in the US remained generally moderate [6].

In the absence of the all-causes death rate, the pulmonary tuberculosis death rate is the next important predictor of *I*_6_. Its inclusion proportion is 55.7%, still well above the threshold of 48.3%. As mentioned in the paper, death rates from pulmonary tuberculosis seem to be a good proxy for the all-causes death rates as they were the highest before the pandemic [7, 8]. The list of selected predictors from the first step analysis is completed by the death rates from cancer and from acute nephritis and Bright’s disease, and the longitude of the city. However, the inclusion proportions of these variables are close to the threshold values, and the variables even become insignificant when the additional 1910 Census variables are involved in the analysis, as shown in **Table A2b**. Note that Acuna-Soto, Viboud and Chowell [6] also revealed for US cities that longitude or latitude coordinates did not affect influenza mortality rates during the pandemic. In addition, **Table A2b** emphasizes a last variable that influences *I*_6_ when included in the simulation without the all-causes death rate, namely the illiteracy rate. Its inclusion proportion of 59.5% is largely above the threshold of 48.8%. The significant effect of the percentage of illiterate residents in a US city on influenza mortality rates and transmissibility has also been highlighted in the research by Grantz et al. [5] and Clay, Lewis and Severnini [3]. Cities with relatively more illiterate residents who could not understand public health messages with pandemic related information experienced a higher level of epidemic explosiveness as measured by *I*_6_.

**Table A2c** presents the final selection models. The model with the all-causes death rate shows that a unit increase in the death rate from organic heart disease, from all causes and the population density increases *I*_6_ by 0.89% (i.e., exp(0.0089) – 1), 0.15% and 1.90%, respectively. Using the means of the predictors (see **Table A1**) as input to this model yields an average prediction for *I*_6_ of 16.661, with location of 2.709 and scale of 0.456. The corresponding lognormal distribution is plotted by the solid line in **Fig A2a**. The model without the all-causes death rate shows that a unit increase in the death rate from organic heart disease and from pulmonary tuberculosis, the population density and illiteracy increases *I*_6_ by 1.17% (i.e., exp(0.0116) – 1), 0.43%, 2.29% and 10.04%, respectively. Using the means of the predictors (see **Table A1**) as input to this model yields an average prediction of 16.535, with location of 2.709 and scale of 0.439. The corresponding lognormal distribution is plotted by the dashed line in **Fig A2a**. The two model distributions largely overlap, with similar prediction performance but slightly more explanatory power of the model without the all-causes death rate.

**Destructiveness variable**

*Pearl’s correlation analysis*

In Study III Pearl conducted another correlation analysis to find associations between the excess mortality rates due to the epidemic in the cities, which he appropriately called the destructiveness of the epidemic, and the same demographic and death rate variables used in Study II. In Study IV Pearl calculated some final correlations between destructiveness and the explosiveness index *I*_6_, for which the zero-order correlation coefficient is 0.709. Although this coefficient is substantial, the left panel of **Table A3a** shows that the zero-order correlation coefficients between destructiveness and the predictor variables are not large and generally smaller than the corresponding coefficients for *I*_6_. The highest correlation of destructiveness is with the normal death rate from organic heart disease. What is striking is that the zero-order coefficient of 0.487 is smaller than the partial coefficient of 0.537 for constant demographic variables. This is mainly due to the high correlation of 0.609 between the heart disease death rate and the age distribution of the population. The older the population, the higher the death rate from organic heart disease and vice versa. A second and last variable that shows a significant association with destructiveness is the normal death rate from all causes. For this variable the partial correlation coefficient of 0.405 for constant demographic variables does not differ all that much from the zero-order coefficient of 0.435. So the usual mortality rates affect not only epidemic explosiveness, but also epidemic destructiveness, or the number of excess deaths during the epidemic. The effect on destructiveness is, however, much smaller than on explosiveness.

*Forward variable selections with a null factor*

Based on **Table A3a** with the forward selections on Pearl’s predictors including and excluding the all-causes death rate in the middle and right panels, we can confirm the prominence of the normal death rate from organic heart disease for explaining epidemic destructiveness. This variable enters the model in most bootstrap replicates (in 69.1% and 77.6% of the replicates with and without the all-causes death rate). Age distribution, population density and the all-causes death rate also seem to have predictive power, but **Table A3b** shows that their effect disappears when we include the additional 1910 Census variables in the analysis. Instead, illiteracy emerges as the second most important variable and replaces the all-causes death rate. The inclusion proportions of illiteracy in the simulations with and without the all-causes death rate are 58.5% and 59.0%, and largely exceed the thresholds of 40.9% and 43.5%. Furthermore, with an inclusion proportion of 49.9%, the share of young adults in the 25-44 age group seems to matter slightly. This result is in line with the relatively high young adult mortality rate due to a combination of influenza and pneumonia in 1918, as illustrated by the age-specific W-shaped mortality curve in the US at that time.

For epidemic destructiveness, the all-causes death rate does not appear to be significant, leaving us with only one final model included in **Table A3c**. This model shows that a unit increase in the death rate from organic heart disease, illiteracy and the share of young adults in the 25-44 age group increases destructiveness by 0.0227, 0.3582 and 0.1853, respectively. Using the means of the predictors (see **Table A1**) as input to this model yields an average prediction of 4.753, with scale of 1.445. The corresponding normal distribution is plotted by the dashed line in **Fig A2b**.

**References**

1. Pearl R. Influenza studies. *Public Health Rep*. 1921;36(7):273-298.
2. Urenda J, Kosheleva O, Kreinovich V, et al. COVID-19 peak immunity values seem to follow log-normal distribution. *Appl Math Sci*. 2020;14(12):599-606.
3. Clay K, Lewis J, Severnini E. What explains cross-city variation in mortality during the 1918 influenza pandemic? Evidence from 438 U.S. cities. *Econ Hum Biol*. 2019;35:42-50.
4. Winslow C-EA, Grove CC. Note on certain correlation factors of the 1918 influenza epidemic. *Am J Hyg*. 1922;2(3):240-245.
5. Grantz KH, Rane MS, Salje H, et al. Disparities in influenza mortality and transmission related to sociodemographic factors within Chicago in the pandemic of 1918. *Proc Natl Acad Sci USA*. 2016;113(48):13839-13844.
6. Acuna-Soto R, Viboud C, Chowell G. Influenza and pneumonia mortality in 66 large cities in the United States in years surrounding the 1918 pandemic. *PLoS One*. 2011;6(8):e23467.
7. Noymer A. Testing the influenza-tuberculosis selective mortality hypothesis with Union army data. *Soc Sci Med*. 2009;68(9):1599-1608.
8. Noymer A. The 1918 influenza pandemic hastened the decline of tuberculosis in the United States: An age, period, cohort analysis. *Vaccine*. 2011;29:B38-B41.

**Table A2a.** **Results for epidemicity index *I*_6_ (Study II) using Pearl’s correlation analysis (left) and bootstrap simulation on the lognormal forward selection model with the null factor and AICc validation (middle and right). Important or selected variables appear in bold on top.**

| Pearl’s Study II | | Forward selection lognormal AICc model simulation | | | |
| --- | --- | --- | --- | --- | --- |
|  |  | DR All causes included (cfr. Pearl) | | DR All causes excluded | |
|  | Correlation coefficient |  | % nonzero in simulation |  | % nonzero in simulation |
| **DR All causes** | **0.678** | **DR Organic heart disease** | **0.871** | **DR Organic heart disease** | **0.964** |
| **DR Organic heart disease** | **0.642** | **DR All causes** | **0.834** | **Population density** | **0.635** |
| DR Pulmonary tuberculosis | 0.578 | **Population density** | **0.748** | **DR Pulmonary tuberculosis** | **0.557** |
| DR Acute nephritis and Bright’s disease | 0.447 | **DR Cancer** | **0.678** | **DR Cancer** | **0.547** |
| DR Typhoid fever | 0.342 | **DR Acute nephritis and Bright’s disease** | **0.588** | **Longitude of city** | **0.523** |
| Sex ratio | -0.307 | NULL FACTOR | 0.511 | NULL FACTOR | 0.453 |
| Percentage pop growth | -0.302 | DR Typhoid fever | 0.480 | DR Typhoid fever | 0.434 |
| Latitude of city | -0.243 | DR Pulmonary tuberculosis | 0.470 | Age distribution | 0.431 |
| DR Cancer | 0.235 | Percentage pop growth | 0.468 | Sex ratio | 0.370 |
| Longitude of city | -0.229 | Age distribution | 0.361 | Percentage pop growth | 0.332 |
| Age distribution | 0.194 | Sex ratio | 0.361 | DR Acute nephritis and Bright’s disease | 0.380 |
| Population density | 0.073 | Latitude of city | 0.328 | Latitude of city | 0.224 |
|  |  | Longitude of city | 0.288 |  |  |
|  |  | **NULL FACTOR 99.9% (Sim) Upper CI** | **0.541** | **NULL FACTOR 99.9% (Sim) Upper CI** | **0.483** |

*Note:* Variables in grey enter about as often as the null factor in the selected models from the 2500 bootstrap replicates. Their inclusion proportions are close to the 99.9% simulated upper confidence limit that is considered as a cut-off.

**Table A2b.** **Extension of the results for epidemicity index *I*_6_ (Study II) using the important variables from Table A2a and additional 1910 Census variables (from Table 2) in bootstrapping the lognormal forward selection model with the null factor and AICc validation. Important variables appear in bold on top.**

| Forward selection lognormal AICc model simulation with additional 1910 Census variables | | | |
| --- | --- | --- | --- |
| DR All causes included (cfr. Pearl) | | DR All causes excluded | |
|  | % nonzero in simulation |  | % nonzero in simulation |
| **DR Organic heart disease** | **0.918** | **DR Organic heart disease** | **0.974** |
| **DR All causes** | **0.760** | **Population density** | **0.636** |
| **Population density** | **0.620** | **DR Pulmonary tuberculosis** | **0.612** |
| NULL FACTOR | 0.446 | **Illiteracy** | **0.595** |
| DR Cancer | 0.422 | Persons to a dwelling | 0.497 |
| DR Acute nephritis and Bright’s disease | 0.420 | Share ages 0-4 | 0.488 |
| Share ages 25-44 | 0.403 | NULL FACTOR | 0.458 |
| Share ages 0-4 | 0.399 | School attendance | 0.392 |
| Persons to a dwelling | 0.397 | Share ages 25-44 | 0.361 |
| Illiteracy | 0.378 | Percentage of homes owned | 0.305 |
| School attendance | 0.277 | DR Cancer | 0.258 |
| Percentage of homes owned | 0.229 | Share ages 65+ | 0.252 |
| Share ages 65+ | 0.220 | Longitude of city | 0.234 |
| **NULL FACTOR 99.9% (Sim) Upper CI** | **0.475** | **NULL FACTOR 99.9% (Sim) Upper CI** | **0.488** |

**Table A2c.** **Selected lognormal regression models for epidemicity index *I*_6_ (Study II) based on the original predictors.**

| Model term | Estimate | Std Error | *P*-value Chi-square test |
| --- | --- | --- | --- |
| DR All causes included (cfr. Pearl) | | | |
| DR Organic heart disease | 0.0089 | 0.0028 | 0.0014 |
| DR All causes | 0.0015 | 0.0005 | 0.0016 |
| Population density | 0.0188 | 0.0103 | 0.0672 |
| Constant | -1.3718 | 0.5994 | 0.0221 |
| Scale lognormal distribution | 0.4559 | 0.0553 | <0.0001 |
| AICc | 239.442 |  |  |
| BIC | 244.931 |  |  |
| Generalized R^2^ | 0.611 |  |  |
| DR All causes excluded | | | |
| DR Organic heart disease | 0.0116 | 0.0028 | <0.0001 |
| Population density | 0.0226 | 0.0099 | 0.0221 |
| DR Pulmonary tuberculosis | 0.0043 | 0.0024 | 0.0745 |
| Illiteracy | 0.0957 | 0.0316 | 0.0024 |
| Constant | -0.7252 | 0.4618 | 0.1163 |
| Scale lognormal distribution | 0.4389 | 0.0532 | <0.0001 |
| AICc | 239.822 |  |  |
| BIC | 245.869 |  |  |
| Generalized R^2^ | 0.640 |  |  |

**Table A3a.** **Results for destructiveness (Study III) using Pearl’s correlation analysis (left) and bootstrap simulation on the normal forward selection model with the null factor and AICc validation (middle and right). Important or selected variables appear in bold on top.**

| Pearl’s Study III | | Forward selection normal AICc model simulation | | | |
| --- | --- | --- | --- | --- | --- |
|  |  | DR All causes included (cfr. Pearl) | | DR All causes excluded | |
|  | Correlation coefficient |  | % nonzero in simulation |  | % nonzero in simulation |
| **DR Organic heart disease** | **0.487** | **DR Organic heart disease** | **0.691** | **DR Organic heart disease** | **0.776** |
| **DR All causes** | **0.435** | **Age distribution** | **0.584** | **Age distribution** | **0.631** |
| DR Pulmonary tuberculosis | 0.428 | **Population density** | **0.499** | **Population density** | **0.477** |
| Latitude of city | -0.325 | **DR All causes** | **0.494** | Latitude of city | 0.420 |
| DR Acute nephritis and Bright’s disease | 0.282 | DR Typhoid fever | 0.448 | NULL FACTOR | 0.405 |
| DR Cancer | 0.215 | NULL FACTOR | 0.436 | DR Pulmonary tuberculosis | 0.368 |
| Population density | 0.111 | Latitude of city | 0.419 | DR Typhoid fever | 0.364 |
| Percentage pop growth | -0.071 | Percentage pop growth | 0.381 | Percentage pop growth | 0.340 |
| Sex ratio | -0.029 | DR Pulmonary tuberculosis | 0.348 | DR Acute nephritis and Bright’s disease | 0.255 |
| Age distribution | 0.024 | DR Acute nephritis and Bright’s disease | 0.334 | Sex ratio | 0.214 |
| DR Typhoid fever | 0.014 | Sex ratio | 0.282 | Longitude of city | 0.201 |
| Longitude of city | 0.001 | DR Cancer | 0.221 | DR Cancer | 0.192 |
|  |  | Longitude of city | 0.203 |  |  |
|  |  | **NULL FACTOR 99.9% (Sim) Upper CI** | **0.465** | **NULL FACTOR 99.9% (Sim) Upper CI** | **0.435** |

*Note:* Variables in grey enter about as often as the null factor in the selected models from the 2500 bootstrap replicates. Their inclusion proportions are close to the 99.9% simulated upper confidence limit that is considered as a cut-off.

**Table A3b.** **Extension of the results for destructiveness (Study III) using the important variables from Table A3a and additional 1910 Census variables (from Table 2) in bootstrapping the normal forward selection model with the null factor and AICc validation. Important variables appear in bold on top.**

| Forward selection normal AICc model simulation with additional 1910 Census variables | | | |
| --- | --- | --- | --- |
| DR All causes and Age distribution included (cfr. Pearl), but no age shares | | DR All causes included (cfr. Pearl) with age shares | |
|  | % nonzero in simulation |  | % nonzero in simulation |
| **DR Organic heart disease** | 0.834 | **DR Organic heart disease** | 0.785 |
| **Illiteracy** | 0.585 | **Illiteracy** | 0.590 |
| DR All causes | 0.429 | **Share ages 25-44** | 0.499 |
| Percentage of homes owned | 0.424 | DR All causes | 0.415 |
| NULL FACTOR | 0.380 | NULL FACTOR | 0.406 |
| Population density | 0.388 | Percentage of homes owned | 0.405 |
| Age distribution | 0.324 | Population density | 0.360 |
| Persons to a dwelling | 0.294 | Share ages 0-4 | 0.342 |
| School attendance | 0.190 | Persons to a dwelling | 0.281 |
|  |  | Share ages 65+ | 0.237 |
|  |  | School attendance | 0.223 |
| **NULL FACTOR 99.9% (Sim) Upper CI** | **0.409** | **NULL FACTOR 99.9% (Sim) Upper CI** | **0.435** |

**Table A3c.** **Selected normal regression model for destructiveness (Study III) based on the original predictors.**

| Model term | Estimate | Std Error | *P*-value *t*-test |
| --- | --- | --- | --- |
| DR Organic heart disease | 0.0227 | 0.0072 | 0.0034 |
| Illiteracy | 0.3582 | 0.1236 | 0.0069 |
| Share ages 25-44 | 0.1853 | 0.1557 | 0.2429 |
| Constant | -7.1643 | 5.4878 | 0.2016 |
| Scale normal distribution | 1.4448 | 0.1931 | <0.0001 |
| AICc | 129.399 |  |  |
| BIC | 134.888 |  |  |
| Generalized R^2^ | 0.408 |  |  |
